# In This Issue

**DOI:** 10.1002/cfg.96

**Published:** 2001-08


**Comparative and Functional Genomics**
*Comp Funct Genom* 2001; **2**: 195.
**DOI:** 10.1002/cfg.96

### In This Issue

### Can Information Extraction technology find evidence of protein interactions from Medline abstracts?

Blaschke and Valencia have applied Information Extraction (IE) technology to the search for protein interaction data in Medline abstracts. They took the 851 interactions detected by the 2-hybrid system from the Database of Interacting Proteins (DIP) and used an IE approach to find evidence for these known interactions. The approach only found evidence to link 30% of the pairs of proteins, and they discuss potential reasons for this.

### Proteomic comparison of three yeast wild type strains: CEN. PK2, FY1679 and W303

Rogowska-Wrzesinska *et al.* present a proteomic study aimed at explaining why a significant number of yeast genes show background strain-specific phenotypes. They found 66 spots on their gels, from which they identified 62 proteins that showed repeatable differences in expression level between the three strains, and they describe some of these in detail.

### SAND: A novel gene family found in *Fugu*, which is conserved across eukaryotes

Cottage *et al.* discovered the SAND gene whilst sequencing a Fugu cosmid. They show that it is conserved in all completely sequenced eukaryotes, from *Saccharomyces cerevisiae* to *Arabidopsis thaliana* to man, and that it appears to have been incorrectly annotated, or completely missed, in three of the genomes. Whilst a battery of bioinformatic analyses are unable to shed any light on the function of this widely conserved family, the group have made a tentative prediction of its tertiary structure.

### Genome-wide generation of yeast gene deletion strains

Kelly *et al.* review the approaches taken by the European Functional Analysis Network (EUROFAN) and the Yeast Gene Deletion Project to make a complete set of mutants for every yeast gene. They report some initial findings, highlight selected studies that have already taken advantage of the mutant collection and discuss future approaches to using this valuable resource.

### Meeting Highlights: CSHL – Genome Sequencing and Biology 2001

We provide an in-depth report on this influential meeting, which covered studies on a range of model organisms, in addition to the more traditional sessions on large scale sequencing, comparative genomics, polymorphism studies and bioinformatics, which were principally concerned with the human genome.

### Meeting Review: The 2$^{nd}$ European Conference on Zebrafish Genetics and Development

Benjamin Feldman reports on this meeting, giving us a snapshot of the progress made in the fields of zebrafish genetics and development, including a report on the strategy to be employed and the progress made so far in the mapping and sequencing of the zebrafish genome.

### Meeting Review: Royal Society Discussion Meeting: Utilising the Genome Sequence of Parasitic Protozoa

The Royal Society recently hosted a public meeting to provide an opportunity for researchers working on a wide range of parasites to discuss and coordinate their work. Neil Hall gives us a taster of this exciting meeting, which covered a range of functional genomics studies.

Copyright © 2001 John Wiley & Sons, Ltd.

